# Application of systems biology to identify pharmacological mechanisms of thrombotic microangiopathy evoked by combined activated prothrombin complex concentrate and emicizumab

**DOI:** 10.1038/s41598-023-36891-x

**Published:** 2023-06-21

**Authors:** Raquel Valls, Jonathan Wagg, Ido Paz-Priel, Gabriel Man, Laura Artigas, Georges Jaccard, Mireia Coma, Christophe Schmitt

**Affiliations:** 1 Anaxomics Biotech SL, Barcelona, Spain; 2 Roche Innovation Center, Basel, Switzerland; 3 Genentech, Inc., South San Francisco, CA USA; 4 Iridessa SA, Saint-Gingolph, Switzerland; 5 F. Hoffmann-La Roche Ltd, Basel, Switzerland; 6 Present Address: AC Immune SA, EPFL Innovation Park, Lausanne, Switzerland; 7 Present Address: Graphite Bio Inc., South San Francisco, CA USA

**Keywords:** Machine learning, Haematological diseases

## Abstract

Emicizumab is a bispecific monoclonal antibody that substitutes for the function of missing or deficient factor VIII (FVIII) in people with hemophilia A (PwHA). Long-term safety and efficacy of emicizumab have been demonstrated in several clinical trials. Nevertheless, in the first of these, three cases of thrombotic microangiopathy (TMA) occurred in PwHA treated with emicizumab receiving high doses of activated prothrombin complex concentrate (aPCC), a bypassing agent used for treating breakthrough bleeds when FVIII neutralizing antibodies (inhibitors) make FVIII replacement ineffective. The aim of the present work is to offer a method to elucidate the pathophysiological and pharmacological mechanisms involved in this treatment-induced TMA. Systems biology and machine learning-based Therapeutic Performance Mapping System is a validated in silico technology that allowed us to construct models of potential mechanisms behind induced TMA. Two drug combinations were modeled and assessed: emicizumab plus aPCC and emicizumab plus recombinant activated factor VII (another bypassing agent). Our models showed that both combinations were related to activation of the coagulation cascade. However, mechanisms involved mainly in platelet activation and possibly in complement activation were detected only for emicizumab plus aPCC, potentially explaining the occurrence of TMA only in this combination.

## Introduction

Hemophilia A is an X-linked, recessive, congenital deficiency of functional plasma clotting factor VIII (FVIII) that presents clinically with bruising, inadequate clotting following injury, and, in severe cases, spontaneous hemorrhaging that can be life-threatening^1,2^. Prophylactic FVIII replacement therapy has been the standard of care for people with hemophilia A (PwHA)^2,3^. However, FVIII preparations have a short half-life, to which is added the burden of three to four intravenous infusions per week. Moreover, around 30% of treated PwHA develop neutralizing alloantibodies (known as inhibitors) that render FVIII replacement therapy ineffective, increasing the risk of serious bleeding and morbidity^4,5^. For those individuals, there are alternative therapies based on prothrombotic coagulation factors that augment other parts of the coagulation cascade, i.e. “bypassing agents”. Bypassing drugs include activated prothrombin complex concentrate (aPCC) and recombinant activated factor VII (rFVIIa)^6–8^. Nevertheless, clinical response to bypassing agents varies among patients^9^.

Emicizumab (Hemlibra) is a subcutaneously administered, recombinant, humanized bispecific monoclonal antibody that bridges activated factor IX (FIXa) and factor X (FX) mimicking the function of missing activated FVIII (FVIIIa), with resultant activation of FX, downstream thrombin generation and activation of the coagulation cascade^10^. Emicizumab has been approved for routine prophylaxis to prevent or reduce the frequency of bleeding episodes in adult and pediatric PwHA with or without FVIII inhibitors^11,12^. The efficacy and safety of emicizumab in a broad population of PwHA have been demonstrated in several clinical trials, showing significantly reduced annualized bleeding rates and beneficial effects on health-related quality of life^11–13^. In pooled phase III clinical trials, thrombotic microangiopathy (TMA) events were reported in less than 1% of total participants^14^. All recorded TMA events, including the three from the HAVEN 1 clinical trial^14^ and a post-marketing case^15^, occurred when, on average, a cumulative amount of more than 100 U/kg/24 h of aPCC was administered for 24 h or more^14,16^.

TMA is a diverse group of disorders characterized by microvascular thrombosis, thrombocytopenia, and microangiopathic hemolytic anemia. The pathophysiology includes endothelial cell and vascular damage that manifests as arteriolar and capillary thrombosis with characteristic abnormalities in the endothelium and vessel wall^17,18^. TMA syndromes can arise from congenital predisposition or from external factors, including infectious or drug-induced TMA (DITMA)^19^. Although some mechanisms of DITMA have been proposed, such as complement activation^20^, for most cases, the underlying mechanisms are unknown. TMA has been rarely reported in PwHA^21^; therefore, its occurrence in participants who had received emicizumab and a concomitant cumulative dose of > 100 U/kg/24 h of aPCC for at least 24 h in the HAVEN 1^14^ trial is unlikely to be a chance occurrence^22^. Moreover, TMA in those individuals, in contrast to the typical clinical course, resolved rapidly following discontinuation of the bypassing agent, aPCC^22^. Nevertheless, the likely underlying mechanisms are not yet clear, and they might be different from the known and proposed DITMA pathophysiological mechanisms^20^. Therefore, a better understanding of these mechanisms may improve mitigation strategies.

The application of in silico and systems biology-based tools may offer a way to generate hypotheses on the specific mechanisms underlying induced TMA in PwHA. Systems biology and machine learning-based methods are increasingly employed to understand drug-evoked responses^23,24^. The utility of this approach has been demonstrated by several artificial intelligence (AI)-based studies across a wide range of complex clinical settings^23–26^. The Therapeutic Performance Mapping System (TPMS) technology is a validated in silico approach^27^ that enables exploration of DITMA’s molecular and cellular mechanisms of action (MoAs). Therefore, we hereby identify and report possible MoAs for how aPCC, when combined with emicizumab, might evoke TMA using this approach.

## Methods

**Figure 1:**
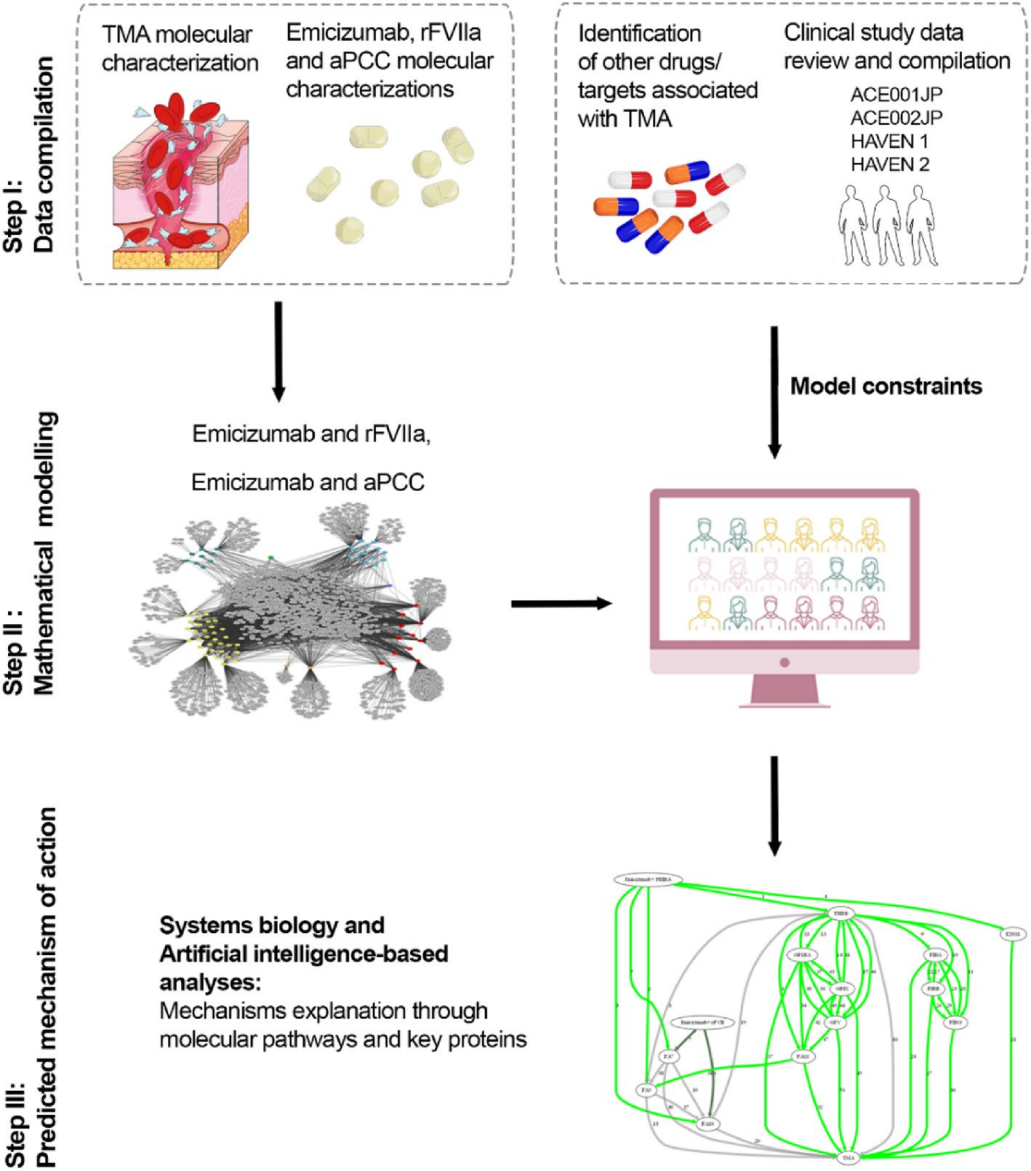
Study workflow. Overview of the in silico approach or our analysis methodology. Figure shows the main steps employed to simulate the MoA of emicizumab in the development of TMA in PwHA, including: (1) data compilation for molecular characterization of TMA and emicizumab, rFVIIa, and aPCC; (2) mathematical model generation using the validated top-down systems biology- and AI-based TPMS approach; and (3) data analyses employing model outputs. *AI* artificial intelligence, *aPCC* activated prothrombin complex concentrate, *MoA* mechanism of action, *PwHA* people with hemophilia A, *rFVIIa* recombinant activated factor VII, *TMA* thrombotic microangiopathy, *TPMS* Therapeutic Performance Mapping Systems.

Figure 1 summarizes the workflow and key steps used to identify candidate pharmacological mechanisms for the clinically observed TMA cases by applying a machine-learning based technology: the TPMS technology previously described^27^. An overview of the Methods for each step is outlined below.

### Data compilation and TMA molecular characterization

It was assumed that DITMA is driven by the pharmacological activation of one or more known pathophysiological TMA mechanisms. Accordingly, the study started by compiling and manually reviewing TMA-related literature to identify all reported TMA pathophysiological mechanisms (referred to as TMA motives) and associated key proteins (referred to as TMA molecular effectors). Relevant scientific articles sourced from the PubMed database (all until January 2017, when the study was performed) were retrieved using the following structured search: (*(thrombotic microangiopathy) OR (thrombotic microangiopathies)) AND (molecular OR protein) AND (pathophysiology)*. Full-length review of these articles identified pathophysiological motives reported to drive TMA (Supplementary Table S1): “Increased platelet activation”, “Increased complement activation”, “Hyperhomocysteinemia”, and “Increased coagulation”. The corresponding molecular effectors playing a biological role in these processes (Supplementary Table S2) were also retrieved from this bibliography review, as well as expanded relevant literature identified through the reference lists derived from the initial set of articles. When a protein candidate was detected in the compiled literature it was included as TMA effector if the reference contained or cited functional evidence of the role of a protein activity change in the causal development of the pathology, to avoid including proteins that might be a marker or reflection of the pathology rather than a causal effector. When the reported evidence for a protein’s role in TMA was deemed insufficient from a functional point of view to be assigned as a TMA effector, additional PubMed searches focused on this protein were completed, and these searches spanned all known protein name synonyms as per UniProtKB codes. Any new TMA molecular effector candidate identified at this stage was subsequently included as TMA effectors following the same criteria applied to review of the initial set of articles. All effectors were grouped by corresponding motives and effectors (Supplementary Tables S1, S2).

### Molecular characterizations for emicizumab, rFVIIa, and aPCC

In a similar fashion, we conducted the identification of all known direct drug targets of key pharmacological agents administered to PwHA during the development of emicizumab. Specifically, molecular characterizations were completed for emicizumab, rFVIIa, and aPCC. A review of dedicated databases (DrugBank^28^, STITCH^29^, SuperTarget^30^) and of relevant scientific literature was performed to identify all known primary drug targets of these agents (Table 1). In the case the agent directly contained the proteins whose functions mediate the studied effect (rFVIIa and aPCC), these proteins were considered, while for drugs inducing a change in activity on other factors, those target factors were considered (i.e. FX activation by emicizumab).

**Table 1:** Summary of the drugs under study characterization at the molecular level.

Drug	DrugBank ID	Protein name	Short name	UniProt	Action
Emi	DB13923	Coagulation factor X	FX	P00742	↑
rFVIIa	DB00036	Coagulation factor VII	FVII	P08709	↑
aPCC	DB13151	Prothrombin	FII	P00734	↑
		Coagulation factor VII	FVII	P08709	↑
		Coagulation factor IX	FIX	P00740	↑
		Coagulation factor X	FX	P00742	↑
		Kininogen-1	KNG1	P01042	↑
		Kallikrein-1 (tissue kallikrein)	KLK1	P06870	↑

The effect of the drug over the proposed target is indicated by an arrow (up = activation).

*aPCC* activated prothrombin complex concentrate, *Emi* emicizumab, *rFVIIa* recombinant activated factor VII.

### Clinical study data review and compilation

Patient-level adverse event profiles for TMA occurrence and other more common emicizumab-associated adverse events were reviewed and compiled to include as part of the training information for the mathematical models describing TMA pathophysiology. Specifically, adverse event data from the emicizumab clinical trials available at the time of this analysis (end of 2017), i.e., ACE001JP^31,32^, ACE002JP^33^, HAVEN 1^14^, and HAVEN 2^34^ were reviewed, compiled, and formatted for TMA model training. All treatment-emergent adverse events were reviewed, but only the subset of events deemed by investigator to be related to emicizumab treatment were selected for further analysis. Subsequently, for each trial, the frequency of all events considered as treatment-related were calculated. For this listing, only the very common adverse drug reactions (occurring with a frequency ≥ 10%), according to the Council for International Organizations of Medical Sciences (CIOMS)^35^, associated with emicizumab were selected for inclusion in the final patient-level adverse event dataset used to train TMA models (Supplementary Table S3).

### Identification of other drugs/targets associated with TMA

PharmaPendium is a proprietary database developed by MDL/Elsevier. The Food and Drug Administration (FDA) provided input under a Cooperative Research and Development Agreement between the FDA and MDL Information Systems. Specifically, PharmaPendium content is derived from multiple information sources including: (i) Searchable FDA Approval Packages; (ii) Meyler’s Side Effect of Drugs; (iii) Drug monographs from Mosby’s Drug Consult; (iv) xPharm target information; (v) Adverse Event Reporting System; (vi) extracted drug metabolite and toxicity data from literature; and (vii) PharmaPendium Historic Files. The resource can be queried by drug (class), by adverse effect/toxicity, by target, and by chemical structure. The “Drug Safety Data” PharmaPendium resource was queried using the term “Thrombotic Microangiopathy” and all retrieved TMA reports exported to an Excel Spreadsheet. A list of all drugs associated with TMA was extracted from these reports (Supplementary Table S4) and enriched with relevant information about known targets, MoAs, and other relevant information from public domain databases, including ChEMBL^36^. This enriched listing of TMA associated drugs/targets was then used as an input dataset for training mathematical models used for TMA mechanism inference.

### TPMS technology: systems biology-based model creation applied to TMA

TPMS is a previously described and validated top-down systems biology approach^23–27^. It is based on AI and pattern recognition techniques that integrate available and relevant biological, pharmacological, and medical knowledge to create mathematical models that are used to simulate in silico clinical responses of interest, in our case, the TMA pathophysiology (Fig. 1).

The protein–protein interaction human network is built using as basic unit nodes the human proteins defined as their UniProtKB SwissProt code, and it incorporates the available relationships (edges or links) between proteins (nodes) from a regularly updated in-house database drawn from public sources, as previously described^23,24,27^. Specific TMA response networks and mechanistic models are constructed using, as starting material, the list of proteins effectors and targets obtained from literature review (Table 1, Supplementary Table S2). Cytoscape software was used for the visualization of molecular interactions defining the TMA response network^37^.

To obtain the mathematical models, the human protein–protein interaction network was trained using AI approaches, as previously extensively described^27^, to comply with a set of known human physiological rules, namely relationships between drugs, defined as their protein targets, and clinical conditions, indications, and adverse drug reactions, defined as proteins involved in the condition (Supplementary Table S5) and specific clinical information on the drugs and TMA response of interest, namely safety outcomes from the above mentioned emicizumab clinical trials and drugs known to induce DITMA (Supplementary Tables S3, S4, respectively). These models were constructed using drug targets (Table 1) as stimulus and the bibliography-based TMA molecular characterization (Supplementary Tables S1, S2) as response. The pharmacological activity (i.e., downstream targets/components) of emicizumab, rFVIIa, and aPCC were characterized and are outlined in Table 1.

As previously described^23,24^, artificial neural networks (ANN) were used to predict associations between the evaluated combination treatments (“emicizumab plus rFVIIa” and “emicizumab plus aPCC”) and characterized TMA mechanisms (motives). The ANN evaluation provides an ANN score per each pair of protein sets evaluated, ranging from 0 to 100%, which provides an idea of the probability that the two sets of proteins are truly related, as per their correspondence to a p-value calculated according to the accuracy against the training set^23,38,39^; these p-values can be used to categorize ANN relationship score results as highly probable of being true (p-value < 0.05), medium probability of being true (0.05 < p-value < 0.25), and low probability of being true (p-value < 0.25). Likewise, sampling-based methods were used to elucidate candidate molecular mechanisms for how these treatments may evoke TMA^23,24^.

### Measures and data analyses MoA model comparison

Common and differential proteins and associated molecular mechanisms predicted to be evoking TMA across the two different combination treatments were identified by systematic analysis of the generated mathematical models. The MoA models of each combination to evoke TMA are a compendium of 100 mathematical solutions that potentially explain how the corresponding treatment may drive TMA. First, the frequency of occurrence of all the interactions within the universe of mathematical solutions included in the MoA model was determined. An optimization algorithm determined the optimal threshold of frequency of occurrence, which allowed simplification of the MoA into a comprehensive (i.e., that presents the interactions and mechanisms most frequent in the universe of solutions) and readable (limiting the number of represented nodes, or proteins) graphical representation. The MoA representation shows whether each interaction frequency is within the threshold for each of the evaluated MoAs.

The impact of each treatment over the activity of the proteins involved in the MoA (either represented or not in the MoA representation) was quantified through the predicted protein activity (ranging from − 1 to 1)^27^. The difference between the predicted protein activity attributed to each protein in the MoA of “emicizumab plus aPCC” and in the MoA of “emicizumab plus rFVIIa” was calculated, and a variation greater than 0.5 (absolute value), was defined as a significant activity change. MoA representation was performed using Graphviz software^40^. Photoshop^41^ program was also employed for assembling multiple-panels figures.

## Results

### TMA pathophysiological network characterization and data compilation

**Figure 2:**
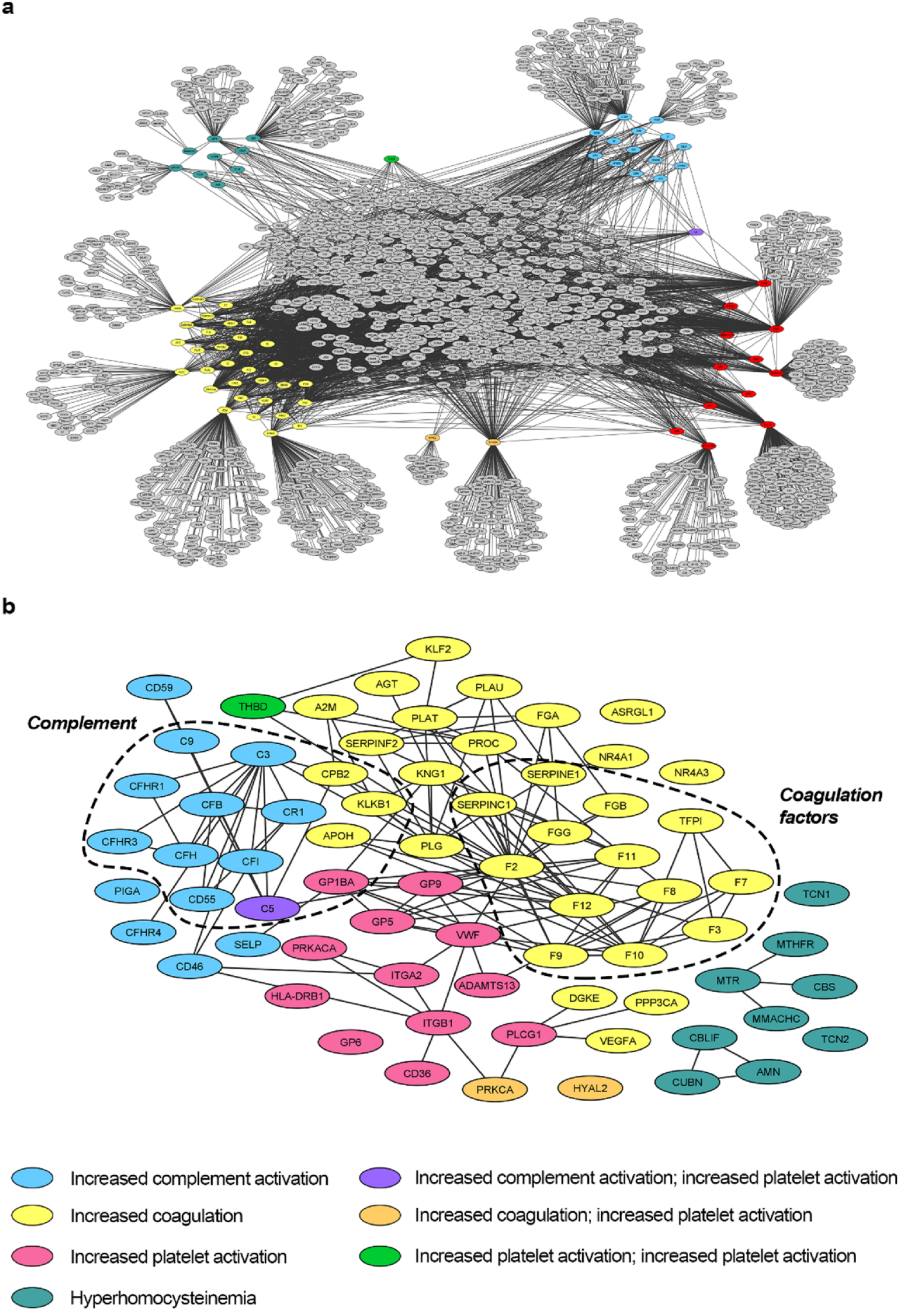
TMA response network. Human protein networks around drugs evoked thrombotic microangiopathy (TMA) molecular pathophysiology considering all disease effectors and their direct interactors. General overview (**a**) and centered on the relationship of TMA response network and overview of TMA effectors and interactions (**b**). Image created with Cytoscape 3.7.0^38^.

Four major TMA pathophysiological mechanisms (motives) were identified: (i) increased platelet activation; (ii) increased complement activation; (iii) hyperhomocysteinemia, and (iv) increased coagulation (Supplementary Table S1). Subsequently, each motive was functionally characterized at the protein level, based on corresponding molecular effectors. A total of 76 distinct TMA protein effectors were identified (Supplementary Table S2) and mapped to one or more corresponding TMA motive. The network around TMA was created by reported interactions (Fig. 2a). Figure 2b provides an overview of the identified effectors and protein interactions between them, grouped by corresponding motives. Note that a small number of identified effectors participate in more than one of the identified motives.

The TMA response network developed around the identified TMA effectors (Fig. 2a) comprises 2199 proteins and 3428 interactions. The resulting network provides a framework for identifying candidate TMA mechanisms as pathways through the network that link known “targets” of the drugs combinations of interest (“emicizumab plus rFVIIa” vs. “emicizumab plus aPCC”) to known TMA effectors.

The PharmaPendium search identified 250 unique drugs/formulations and 323 drug targets associated with one or more reports of TMA association. For this analysis, a threshold of five or more reports was established to identify the subset of these drugs/formulations and associated targets for which there was consistent evidence of TMA association. This threshold identified 71 distinct drugs/formulations and 115 associated targets, which were subsequently used as an input data set for training the TMA network model (Supplementary Table S4).

The patient-level adverse event profiles of emicizumab clinical studies were reviewed and compiled. The reported cases of TMA occurred in the HAVEN 1 study population. At the time of analysis (end of 2017), a total of: (i) 225 adverse event occurrences (125 uniquely named events) were reported across 75 participants in HAVEN 1; (ii) 43 adverse event occurrences (24 uniquely named events) were reported across 14 participants in HAVEN 2; (iii) 72 adverse event occurrences (46 uniquely named events) were reported across 33 participants in ACE001JP; (iv) 253 adverse event occurrences (104 uniquely named events) were reported across 18 participants in ACE002JP. The most common (very common according to the CIOMS; frequency > 10%) treatment-related adverse events in one or more of the four trials were injection-site reactions, upper respiratory tract infection, and headache (see Supplementary Table S3 online for summary of adverse event profiles across these studies). Accordingly, these three events, in addition to the rarer cases of TMA, were included as model restrictions, i.e., used to train TMA models.

### Effect of the coadministration of “emicizumab plus rFVIIa” vs. “emicizumab plus aPCC” on known TMA mechanisms and associated effectors

Following training, the TMA model was used to assess the likely impact of the coadministration of “emicizumab plus rFVIIa” vs. “emicizumab plus aPCC” on known TMA mechanisms and associated effectors. The relationships of each drug target with TMA main pathophysiological motives were evaluated by the ANN (Table 2). The ANN analysis showed that, in general, both combination therapies have a potential relationship with TMA (p < 0.05), according to the molecular characterization used. When looking at specific pathophysiological motives, both combinations were predicted to trigger the coagulation cascade (p < 0.05). While no other motive was found to present a high probability relationship with either of the combinations (p < 0.05), “emicizumab plus aPCC” was predicted to be qualitatively more related to increased complement activation than “emicizumab plus rFVIIa” (50.1% vs. 5.09%; p < 0.25 vs. p > 0.25). Platelet activation also showed some differences in predicted ANN score among the combinations (37.33% vs. 14.36%), although both fell on the same low probability of relationship category (p > 0.25).

**Table 2:** Relationship of “emicizumab plus rFVIIa” and “emicizumab plus aPCC” targets and pathophysiological motives calculated by artificial neural networks.

Pathophysiological processes (motives)	Emi + rFVIIa ANN category (ANN score)	Emi + aPCC ANN category (ANN score)
TMA	+++ (94.42%)	+++ (91.96%)
Hyperhomocysteinemia	+ (16.67%)	+ (10.21%)
Increased coagulation	+++ (85.2%)	+++ (77.52%)
Increased complement activation	+ (5.09%)	++ (50.1%)
Increased platelet activation	+ (14.36%)	+ (37.33%)

The percentage values correspond to the ANN score. Categories: High (+++) corresponds to p-value < 0.05 (ANN score [100,77]); Medium (++) corresponds to p-value < 0.25 (ANN score (77,38]); Low (+) correspond to p-value > 0.25 (ANN score < 38).

*ANN* artificial neural networks, *aPCC* activated prothrombin complex concentrate, *Emi* emicizumab, *rFVIIa* recombinant activated factor VII, *TMA* thrombotic microangiopathy.

**Figure 3:**
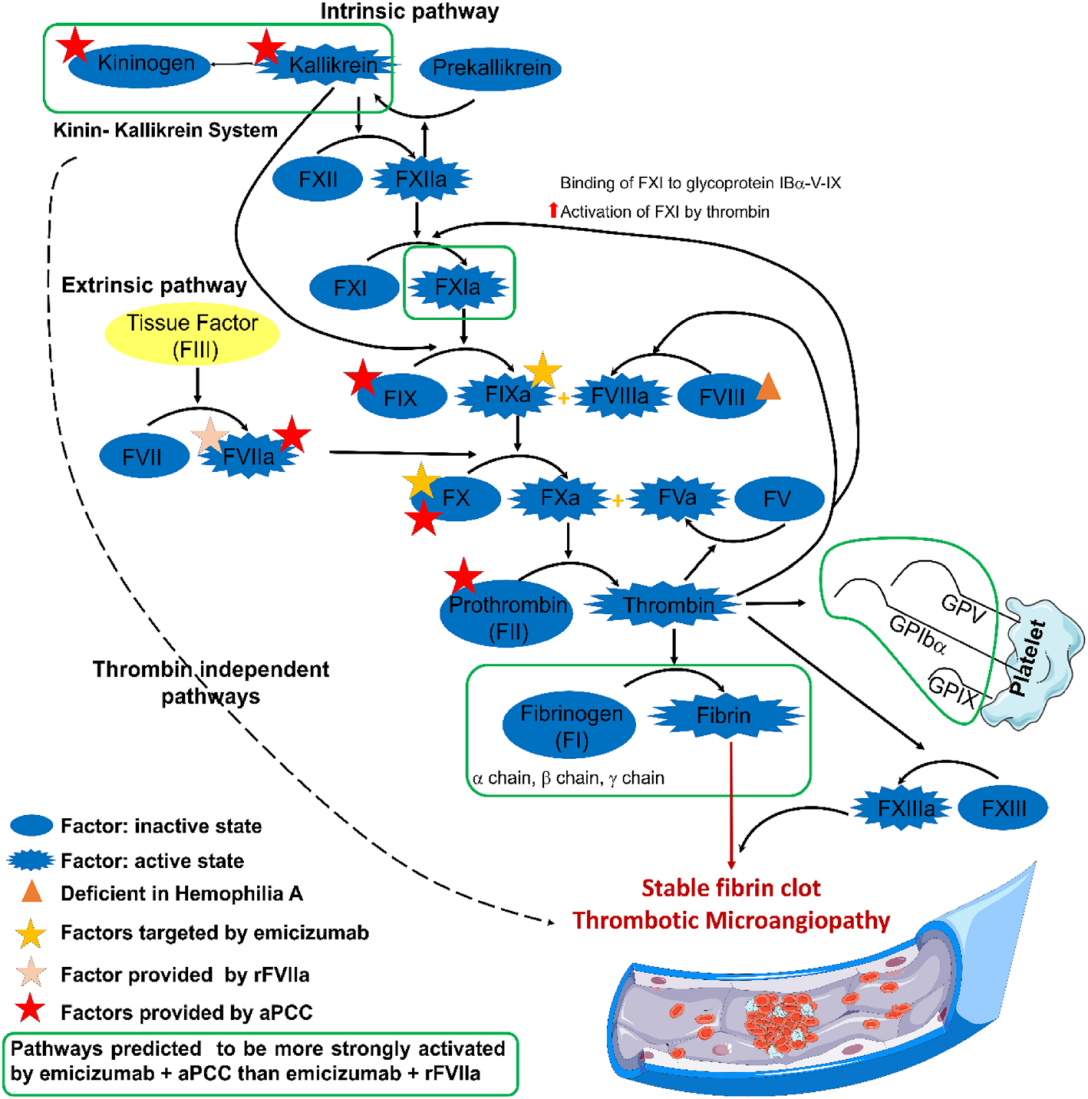
Mechanisms of action of emicizumab, rFVIIa, and aPCC DITMA. Overview of the mechanism of action in terms of major proteins and associated pathways linking “emicizumab plus rFVIIa” or “emicizumab plus aPCC” direct interactions to downstream characterized TMA effectors. Green lines indicate mechanisms triggered by “emicizumab plus aPCC” but not detected for “emicizumab plus rFVIIa” according to the models. Supplementary Fig. S1 and Supplementary Table S6 contain the sources of information found in the scientific literature supporting the predicted mechanisms. *a* activated, *aPCC* activated prothrombin complex concentrate, *DITMA* drug-induced TMA, *F* factor, *rFVIIa* recombinant activated factor VII, *TMA* thrombotic microangiopathy.

As in the ANN evaluation, sampling-based methods highlighted a key role for both combinations in activating coagulation (Fig. 3, Supplementary Fig. S1, Supplementary Table S6); both combinations can modulate the activity of key coagulation factors (FVII, FIX, FX, and prothrombin). However, the models predict a more extensive role for “emicizumab plus aPCC” in mechanisms inducing TMA, which suggests a potential explanation for “emicizumab plus aPCC” DITMA. Specifically, the “emicizumab plus aPCC” combination is predicted to activate the following additional TMA-related mechanisms more strongly: (i) the platelet glycoprotein Ib-V-IX (GP1bα, GPV, and GPIX) major adhesive receptor; and (ii) the fibrinogenesis process (fibrinogen α, β, and γ chain). Moreover, assuming trace amounts of pharmacologically active kallikrein (KLK1) and kininogen (KNG1) in aPCC composition, the model highlights the role of this system in the intrinsic coagulation pathways as potentially contributing to “emicizumab plus aPCC” DITMA.

**Figure 4:**
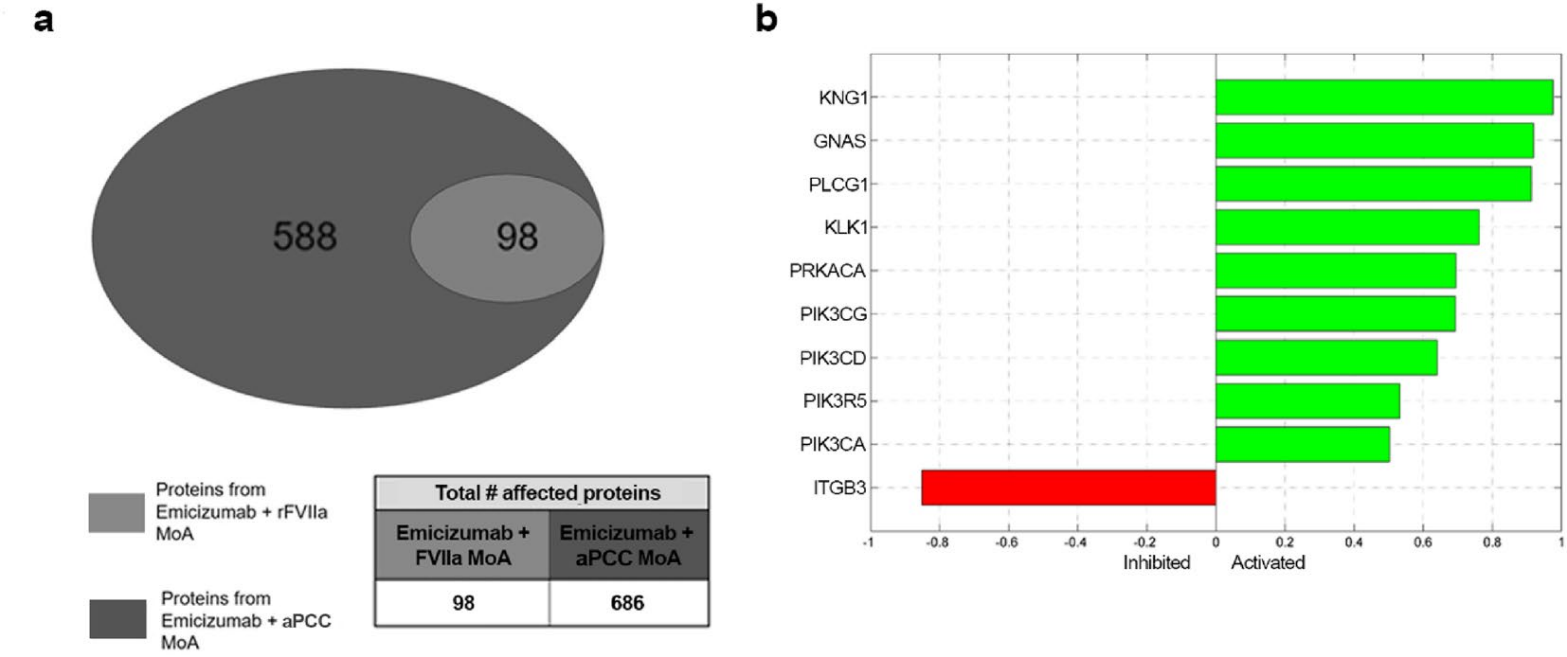
Overview of the main proteins whose predicted differential activity levels are predicted to differentiate the effects of “emicizumab plus aPCC” vs. those of “emicizumab plus rFVIIa”. (**a**) Number of proteins whose functions are predicted to be perturbed by the combination of “emicizumab plus rFVIIa” vs. “emicizumab plus aPCC”. (**b**) Differential predicted protein activity of proteins that allow to differentiate the effects of “emicizumab plus aPCC” vs. those of “emicizumab plus rFVIIa”; Inhibited proteins indicate an inhibition in “emicizumab plus aPCC” respect “emicizumab plus rFVIIa”, Activated proteins indicate an activation in “emicizumab plus aPCC” respect “emicizumab plus rFVIIa”. *aPCC* activated prothrombin complex concentrate, *FVIIa* activated factor VII, *GNAS* adenylate cyclase-stimulating G alpha protein, *ITGB3* integrin beta-3, *KLK1* kallikrein, *KNG1* kininogen, *MoA* mechanism of action, *PIK3CA* phosphatidylinositol 3-kinase catalytic, alpha, *PIK3CD* phosphatidylinositol 3-kinase catalytic, gamma, *PIK3CG* phosphatidylinositol 3-kinase catalytic, gamma, *PIK3R5* phosphoinositide-3-kinase, regulatory subunit 5, *PLCG1* phospholipase C, gamma 1, *PRKACA* cAMP-dependent protein kinase catalytic subunit alpha, *rFVIIa* recombinant activated factor VII.

To evaluate the extent of the difference between the MoAs of both combinations, we explored the complete pool of mechanistic solutions, and found that all the candidate mechanisms of TMA modulated by “emicizumab plus rFVIIa” were also be triggered by “emicizumab plus aPCC” (full overlap), while the latter combination evokes additional candidate TMA mechanisms (Fig. 4a). Specifically, “emicizumab plus rFVIIa” is predicted to indirectly drive changes across 98 distinct TMA network proteins, whilst the combination of “emicizumab plus aPCC” is predicted to drive changes across a wider range of 686 TMA network proteins, including the 98 modulated by “emicizumab plus rFVIIa” (Supplementary Table S7).

Furthermore, the model-inferred MoAs of “emicizumab plus aPCC” vs. “emicizumab plus rFVIIa” were compared to identify the model-predicted protein activity levels that most differed between the MoA models (Fig. 4b). In line with previous results, there was a stronger modulation of the predicted activity of proteins known to drive platelet signaling and activation with “emicizumab plus aPCC”. These proteins were (Fig. 4b): integrin beta-3 (ITGB3)—the activity of which is reduced in “emicizumab plus aPCC” with respect to “emicizumab plus rFVIIa”—, phospholipase C, gamma 1 (PLCG1), cAMP-dependent protein kinase catalytic subunit alpha (PRKACA), phosphatidylinositol 3-kinase catalytic, alpha (PK3CA), phosphatidylinositol 3-kinase catalytic, delta (PK3CD), phosphatidylinositol 3-kinase catalytic, gamma (PK3CG), phosphoinositide-3-kinase, regulatory subunit 5 (PI3R5), and adenylate cyclase-stimulating G alpha protein (GNAS2)—whose activity is upregulated in “emicizumab plus aPCC” vs. “emicizumab plus rFVIIa”. Also, according to the assumption of presence of trace amounts of pharmacologically active KLK1 and KNG1 in aPCC, the activity of these two kinin system proteins was predicted to be upregulated by the “emicizumab plus aPCC”.

## Discussion

TMA was reported only after the concomitant administration of a cumulative dose of > 100 U/kg/24 h for at least 24 h of aPCC in three emicizumab-treated PwHA with FVIII inhibitors^14,22^, but not with co-exposure to emicizumab and rFVIIa^42^. In the present work, we used a systems biology approach to propose plausible hypotheses on the candidate molecular mechanisms triggering TMA after the administration of emicizumab in combination with aPCC. We identified that combination of both aPCC and rFVIIa with emicizumab are related to the activation of the coagulation cascade (as expected due to their therapeutic uses), and propose that other processes (i.e., complement and platelet activation) are associated with aPCC treatment and might explain the occurrence of DITMA in some PwHA treated with emicizumab plus aPCC.

Our mechanistic analysis identified several MoAs that might explain the development of TMA in the studied clinical conditions. The coagulation cascade is triggered by both drug combinations, through activation of FVII, FIX, FX, and prothrombin^43^, which is in line with the therapeutic effect of both combinations in treated PwHA. However, while our models are able to detect the existence of a relationship for both combinations with the activation of the coagulation cascade, in vitro studies done to date suggest a quantitative difference in the ability of each bypassing agent to activate this cascade, which might arise from the different factors affected by each bypassing agent and consequent mechanism of action: the combination of aPCC (0.5 U/mL) and emicizumab (sequence-identical analog, 600 nM [~ 87 µg/mL]) results in a 17-fold increase in thrombin generation over emicizumab alone (above normal thrombin levels), in contrast with coadministration of rFVIIa (1.75 µg/mL) which produced only a 1.8-fold increase^44^. This over-activation induced by aPCC cannot be detected by our models, which already detected optimal effect of both combinations over this pathway, according to their therapeutic potential. This difference in thrombin activation was attributed to the presence of FIX and FX in aPCC^44^, one of the target antigens of emicizumab, which contributes to a synergistic increase in thrombin generation evoked by emicizumab plus aPCC. Interestingly, in vitro studies with Mim8 (another anti FIXa/FX bispecific antibody) also show that the FIXa present in aPCC appears to be the reason for excessive thrombin generation^45^. FIX appears involved in the mechanisms linking aPCC to TMA development, according to our models. This synergistic effect, added to the high cumulative aPCC doses observed in the patients that developed DITMA, might have led to excessive coagulation activation, and could have contributed to DITMA occurrence.

Beside the direct effect on the coagulation cascade, the combination “emicizumab plus aPCC” in our analysis might additionally increase complement and platelet activation pathways, which are potential MoAs for TMA. Our ANN results propose a possible relationship with complement activation and “emicizumab + aPCC”, with higher score than “emicizumab + rFVIIa”. However, the mechanisms linking “emicizumab + aPCC” with the complement system are unclear, since the obtained score has a modest statistical significance (p-value < 0.25), and the application of sampling methods did not highlight any specific pathway. However, a recent report by Tsai et al. reported a case of aPCC-induced atypical hemolytic uremic syndrome which was successfully treated with the anti-C5 antibody eculizumab, thereby relating aPCC with terminal activation of the complement system^46^; further studies might clarify this potential relationship. Regarding platelet activation, and beside its effect through increase of generation of thrombin as shown in vitro^44^, this drug combination activated the fibrinogenesis process more strongly^47^. Activation of the glycoprotein Ib-V-IX major adhesion receptor is also increased by “emicizumab plus aPCC” in a greater degree, and thrombin binding to glycoprotein Ib-V-IX has been demonstrated to induce platelet adhesion and aggregation^47^. Finally, the model-based results indicate that a modulation of the KNG1 pathway by the “emicizumab plus aPCC” combination could be related to TMA, since KNG1 is an initiator of the intrinsic coagulation pathway^48^. However, while detectable, it remains uncertain as to whether the trace quantities of kallikrein–kinin system agents present in aPCC were high enough to drive TMA-related processes. In-depth models analysis showed that the “emicizumab plus aPCC” combination also differentially modulates PLCG1 and KAPCA, two known players in TMA^49^. Additionally, modulation of the phosphatidylinositol 3-kinase (PI3K) signaling pathway might contribute to thrombus formation by platelet activation^50^ and alter TMA-associated microvascular repair^51^. Our models also show that the “emicizumab plus aPCC” combination reduces ITGB3; knockout experiments showed a role for ITGB3 expression disruption in TMA development^52^. These additional mechanisms might be potential new discoveries in the DITMA field. The last process evaluated, hyperhomocysteinemia, while being reported to be associated to TMA in other settings^53^ has not been shown in our results as being part of the TMA mechanisms induced by “emicizumab plus aPCC”. The mechanisms linking hyperhomocysteinemia with TMA, while not fully understood, involve abnormal red cell and platelet production and endothelial dysfunction^54^ which, although ultimately linked to the existence of a hypercoagulable state^55,56^, might affect upstream of the mechanisms predicted for “emicizumab plus aPCC”.

Our models point towards alternative mechanisms induced by “emicizumab plus aPCC”, although there is some overlap on mechanisms also induced by “emicizumab plus rFVIIa” (98 proteins out of 588). However, these shared mechanisms do not necessarily share the same pattern of activation, i.e., the extent of activation may differ across these two combinations, since the additional aPCC components, or high doses of the treatment, as occurred in the observed clinical cases, may modify the effects driven solely by rFVIIa. These results, consistent with the fact that FVIIa is also a component of aPCC, point towards the irrelevance of rFVIIa coadministration as a major TMA driver.

Discontinuation of aPCC resulted in resolution of TMA, highlighting the fact that emicizumab monotherapy is not sufficient to cause DITMA^20^. Emicizumab continued to demonstrate a favorable safety profile, and no deaths or TMA occurrence were reported in subsequent clinical trials^13^. In the post-marketing setting, TMA has been only identified in one additional patient treated with emicizumab and high dose aPCC (> 100 U/kg/24 h; doses beyond label warning^11^) from over 20,000 PwHA treated with emicizumab (global exposure as of April 2023)^15,16,22,57^. Moreover, ex vivo experiments have shown that concomitant use of rFVIIa or aPCC with emicizumab did not mediate excessive thrombus formation and remains an option for the hemostatic management of PwHA with FVIII inhibitors^58^. Therefore, clinical data indicates that aPCC can possibly be used safely with emicizumab, although caution must be exercised around dosing of aPCC^11^. Nevertheless, it would be important to explore the specific mechanistic hypotheses reported herein. Our insights into the underlying mechanisms involved in DITMA may be used to inform the design of targeted experimental studies, which will further assess the specific effects of “emicizumab plus aPCC” on the activities of the proteins identified explicitly as modified by this combination, and how these changes mechanistically drive TMA.

As with previous work in other clinical conditions^23–27^, our in silico study confirms the ability of systems biology approaches to suggest mechanistic insights on therapies and disease MoAs, but it has some limitations. Our models and their biological validation are limited to the available data on TMA, hemophilia A, and drugs available in repositories, and the scientific knowledge at the time of the study. New studies have been carried out since the start of this study (we only included molecular information until 2017, and clinical studies reported at that time: ACE001JP, ACE002JP, HAVEN 1, and HAVEN 2), and future studies may increase the body of relevant clinical and physiological data. The inclusion of any such new data into the model-based analysis might yield more accurate results. Moreover, our technique employed a qualitative modeling approach, which does not consider quantitative information such as drug dosing and constituting factor amounts. Therefore, the quantitative impacts or contribution of the identified factors would not be evaluated. Nevertheless, the TPMS technology builds models considering the whole human protein network and a wide range of drug–pathology relationships (Supplementary Table S5)^27^, not only limited to TMA or hematologic indications, and the models present cross-validation accuracies above 80% in the case of ANN models and above 90% in sampling methods-based models^27^. Having proposed possible MoAs underlying “emicizumab plus aPCC”-evoked TMA, we suggest to experimentally explore them to further understand the underlying DITMA MoA.

In conclusion, our systems biology study agrees with the clinical data showing that the combination of emicizumab and aPCC at high doses (> 100 U/kg/24 h; doses beyond label warning^11^), but not rFVIIa, is plausibly the primary driver of the TMA events recorded in emicizumab-treated PwHA. Our systems biology study identified the differential activation of mechanism by each of these combinations, thereby suggesting potential driving motives for this novel, rare phenomenon. For these patients, emicizumab is a viable therapeutic option. Although bypassing agents are indicated for both prophylaxis and bleeding episodes, the use of more than 100 U/kg/24 h of aPCC for 24 h or more for PwHA on emicizumab should be avoided^11^, and rFVIIa should be the first option used to treat bleeds. The present work, which offers an in silico modeling of the TMA pathways triggered by the combined used of emicizumab and high dose aPCC, sheds some light upon the possible pathophysiological mechanisms involved.

## Supplementary Information

Supplementary Information.

## Data Availability

The authors confirm that the data supporting the findings of this study are available within the article and its Supplementary Materials.
