# Supplementary material for: Application of systems biology to identify pharmacological mechanisms of thrombotic microangiopathy evoked by combined activated prothrombin complex concentrate and emicizumab
Source: Sci Rep. 2023 Jun 21;13:10078. doi: 10.1038/s41598-023-36891-x (PMC10284911; doi:10.1038/s41598-023-36891-x)
Supplement: Supplementary file 1 — Supplementary Information. [file 41598_2023_36891_MOESM1_ESM.pdf]

Supplementary tables and figures

Supplementary Figure S1. Overview of the mechanism of action of “emicizumab plus rFVIIa” or “emicizumab plus aPCC” evoked TMA.

Figure created to represent TPMS MoA predictions using Graphviz software. All links have been manually reviewed: the link number corresponds to the reference code in Supplementary Table S6 online, which contains the sources of information found in the scientific literature supporting the predicted mechanisms. Green arrows show activation; red lines show inhibition; broken-lined circles indicate a node that contains more than one protein, all acting in the MoA in the same way. Gene name is used for protein depiction. aPCC: activated prothrombin complex concentrate, MoA: mechanism of action, rFVIIa: recombinant activated factor VII, TMA: thrombotic microangiopathy, TPMS: Therapeutic Performance Mapping Systems.

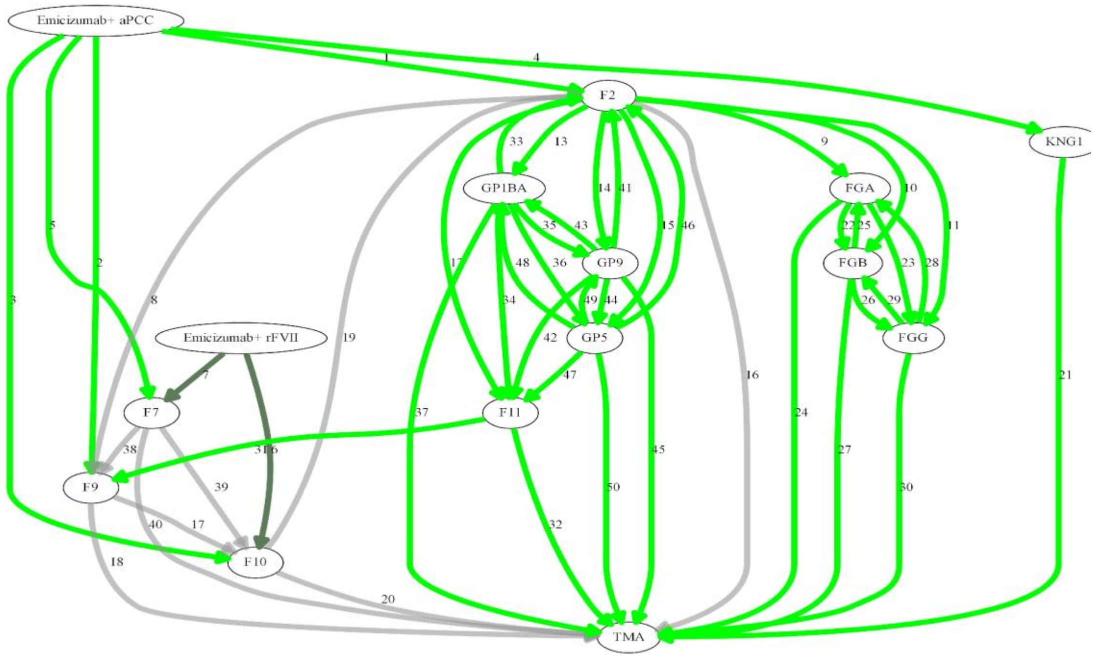

Supplementary Table S1: Summary of the motives identified as involved in TMA.

The amount of protein effectors identified in each motive are noted.

Pathophysiological motive	# Proteins
Increased platelet activation	15
Increased complement activation	17
Hyperhomocysteinemia	9
Increased coagulation	35
Total non-duplicated proteins	71

Supplementary Table S2. Proteins included in the TMA molecular characterization.

Activation in TMA column indicates whether the protein is increased/overactivated (1) or reduced/inhibited (-1) in TMA. Note that proteins can be repeated in more than one motive.

MOTIVE NAME	Effector Protein (Name)	Effector Protein (Short Name)	Effector Protein (GeneName)	Effector Protein (Uniprot)	Activation in TMA (1, -1)	Reference number	Reference
Increased platelet activation	A disintegrin and metalloproteinase with thrombospondin motifs 13	ADAMTS13	ATS13	Q76LX8	-1	1	PMID: 16388413, PMID: 20058209, PMID: 17666281, PMID: 16131569
Increased platelet activation	von willebran factor	VWF	VWF	P04275	1	2	PMID: 21816831
Increased platelet activation	Platelet glycoprotein 4	CD36	CD36	P16671	-1	3	PMID: 7529543, PMID: 19389207
Increased platelet activation	cAMP-dependent protein kinase catalytic subunit alpha	PRKACA	KAPCA	P17612	1	4	PMID: 21816831
Increased platelet activation	1-phosphatidylinositol 4,5-bisphosphate phosphodiesterase gamma-1	PLCG1	PLCG1	P19174	1	5	PMID: 21816831
Increased platelet activation	Protein kinase C alpha type	PRKCA	KPCA	P17252	1	6	PMID: 21816831
Increased platelet activation	Integrin alpha-2 (glycoprotein Ia)	ITGA2	ITA2	P17301	1	7	Nigel Key; Michael Makris; et al. (2009). Practical Hemostasis and Thrombosis. Wiley-Blackwell. p.

							2. ISBN 978-1-4051-8460-1.
Increased platelet activation	Integrin beta-1 (glycoprotein IIa)	ITGB1	ITB1	P05556	1	8	Nigel Key; Michael Makris; et al. (2009). Practical Hemostasis and Thrombosis. Wiley-Blackwell. p. 2. ISBN 978-1-4051-8460-1.
Increased platelet activation	Platelet glycoprotein Ib alpha chain	GP1BA	GP1BA	P07359	1	9	Nigel Key; Michael Makris; et al. (2009). Practical Hemostasis and Thrombosis. Wiley-Blackwell. p. 2. ISBN 978-1-4051-8460-1.
Increased platelet activation	Platelet glycoprotein IX	GP9	GPIX	P14770	1	10	Nigel Key; Michael Makris; et al. (2009). Practical Hemostasis and Thrombosis. Wiley-Blackwell. p. 2. ISBN 978-1-4051-8460-1.
Increased platelet activation	Platelet glycoprotein V	GP5	GPV	P40197	1	11	Nigel Key; Michael Makris; et al. (2009). Practical Hemostasis and Thrombosis. Wiley-Blackwell. p. 2. ISBN 978-1-4051-8460-1.

Increased platelet activation	Platelet glycoprotein VI	GP6	GPVI	Q9HCN6	1	12	Nigel Key; Michael Makris; et al. (2009). Practical Hemostasis and Thrombosis. Wiley-Blackwell. p. 2. ISBN 978-1-4051-8460-1.
Increased platelet activation	Complement C5	C5	CO5	P01031	1	13	PMID: 18503589
Increased platelet activation	HLA class II histocompatibility antigen, DRB1-14 beta chain	HLA-DRB1	2B1E	Q9GIY3	1	14	PMID: 26146809
Increased platelet activation	Hyaluronidase-2	HYAL2	HYAL2	Q12891	-1	15	PMID: 25934767
Increased complement activation	Complement receptor type 1	CR1	CR1	P17927	-1	16	PMID: 2581636 and PMID: 2956607
Increased complement activation	Complement decay-accelerating factor	CD55	DAF	P08174	-1	17	PMID: 2581636 and PMID: 2956607
Increased complement activation	Membrane Cofactor Protein	CD46	MCP	P15529	-1	18	PMID: 14615110, PMID: 14566051, PMID: 23635876
Increased complement activation	Complement Factor H	CFH	CFAH	P08603	-1	19	PMID: 2956607, PMID: 16528247, PMID: 2956607
Increased complement activation	Complement factor I	CFI	CFAI	P05156	-1	20	PMID: 15917334
Increased complement activation	Complement C3	C3	CO3	P01024	1	21	PMID: 18796626, Charles A Janeway, J., Paul Travers, Mark Walport, and Mark J Shlomchik, Immunobiology, The Immune

							System in Health and Disease. 2001.
Increased complement activation	Complement Factor B	CFB	CFAB	P00751	1	22	PMID: 17182750
Increased complement activation	Thrombomodulin	THBD	TRBM	P07204	-1	23	PMID: 19625716
Increased complement activation	Complement factor H-related protein 1	CFHR1	FHR1	Q03591	-1	24	PMID: 24333077, PMID: 19861685 and PMID: 19625716
Increased complement activation	Complement factor H-related protein 3	CFHR3	FHR3	Q02985	-1	25	PMID: 24333077, PMID: 19861685 and PMID: 19625716
Increased complement activation	Complement factor H-related protein 4	CFHR4	FHR4	Q92496	-1	26	PMID: 24333077, PMID: 19861685 and PMID: 19625716
Increased complement activation	Phosphatidylinositol N-acetylglucosaminyltransferase subunit A	PIGA	PIGA	P37287	-1	27	PMID: 19707355
Increased complement activation	P-selectin	SELP	LYAM3	P16109	1	28	PMID: 21642543
Increased complement activation	Complement factor B	CFB	CFAB	P00751	1	29	PMID: 17182750

Increased complement activation	Complement C9	C9	CO9	P02748	1	30	Charles A Janeway, J., Paul Travers, Mark Walport, and Mark J Shlomchik, Immunobiology, The Immune System in Health and Disease. 2001.
Increased complement activation	Complement C5	C5	CO5	P01031	1	31	[1] Terminal Complement Inhibitor Eculizumab Improves Complement-Mediated Platelet Consumption and Thrombocytopenia in Patients with Paroxysmal Nocturnal Hemoglobinuria (PNH). Gerard Socie, Petra Muus, Hubert Schrezenmeier, Britta Höchsmann, Jaroslaw P. Maciejewski, Ilene Ceil Weitz, Anita Hill, Monica Bessler and Antonio M. Risitano Blood 2009 114:4030; [2] Charles A Janeway, J., Paul Travers, Mark

							Walport, and Mark J Shlomchik, Immunobiology, The Immune System in Health and Disease. 2001.
Increased complement activation	CD59 glycoprotein	CD59	CD59	P13987	-1	32	PMID: 1372164
Hyperhomocysteinaemia	Cystathionine beta-synthase	CBS	CBS	P35520	-1	33	PMID: 11011842
Hyperhomocysteinaemia	Methionine synthase	MTR	METH	Q99707	-1	34	Homocystinuria Caused by Cystathionine Beta-Synthase Deficiency. Chapter.Jonathan D Picker, MBChB, PhD and Harvey L Levy, MD. Last Update: November 13, 2014. Source: PubMed. In book: GeneReviews™
Hyperhomocysteinaemia	Cubilin	CUBN	CUBN	O60494	-1	35	PMID: 16722557
Hyperhomocysteinaemia	Protein amnionless	AMN	AMNLS	Q9BXJ7	-1	36	PMID: 16722557
Hyperhomocysteinaemia	Transcobalamin-2	TCN2	TCO2	P20062	-1	37	PMID: 16722557, PMID: 1743216

Hyperhomocysteinaemia	Transcobalamin-1	TCN1	TCO1	P20061	-1	38	PMID: 16722557 and PMID: 1743216
Hyperhomocysteinaemia	Gastric intrinsic factor	GIF	IF	P27352	-1	39	PMID: 16722557 and PMID: 1743216
Hyperhomocysteinaemia	Methylmalonic aciduria and homocystinuria type C protein	MMACHC	MMAC	Q9Y4U1	-1	40	PMID: 12210350, PMID: 15754282
Hyperhomocysteinaemia	Methylenetetrahydrofolate reductase	MTHFR	MTHR	P42898	-1	41	PMID: 15897349
Increased coagulation	Thrombomodulin	THBD	TRBM	P07204	-1	42	PMID: 1848730, PMID: 12920633, PMID: 9399073
Increased coagulation	Plasminogen	PLG	PLMN	P00747	-1	43	PMID: 24029428, PMID: 9399073
Increased coagulation	diacylglycerol kinase-ε	DGKE	DGKE	P52429	-1	44	PMID: 23542698, PMID: 23619787
Increased coagulation	Protein kinase C	PRKCA	KPCA	P17252	1	45	PMID: 23619787
Increased coagulation	Vitamin K-dependent protein C	PROC	PROC	P04070	-1	46	PMID: 1848730, PMID: 24029428
Increased coagulation	Vascular endothelial growth factor A	VEGFA	VEGFA	P15692	-1	47	PMID: 18337603 ,PMID: 21086498
Increased coagulation	Alpha-2-macroglobulin	A2M	A2MG	P01023	-1	48	PMID: 1758145
Increased coagulation	alpha-2-antiplasmin	SERPINF2	A2AP	P08697	-1	49	PMID: 1758145
Increased coagulation	Calcineurin	PPP3CA	PP2BA	Q08209	-1	50	PMID: 25414441, PMID: 1848730
Increased coagulation	Carboxypeptidase B2	CPB2	CBPB2	Q96IY4	-1	51	PMID: 20088943, PMID: 17327284, Toshihiko Nishimura, et al., Carboxypeptidase B2 Is Protective in a Mouse Model of Shiga Toxin-Induced Hemolytic Uremic Syndrome,

							Blood 2014 124:2804
Increased coagulation	beta2 glycoprotein 1	APOH	APOH	P02749	-1	52	[1] PMID: 21385543, [2] Info provided by Jon [3] Info provided by Jon [4] Info provided by Jon [5] Info provided by Jon[6]Info provided by Jon [7] PMID: 8611459 [8] PMID: 4052628
Increased coagulation	Isoaspartyl peptidase/L-asparaginase	ASRGL1	ASGL1	Q7L266	1	53	PMID: 7725851
Increased coagulation	Krueppel-like factor 2	KLF2	KLF2	Q9Y5W3	-1	54	PMID: 15718498, PMID: 25185079 and PMID: 15718498
Increased coagulation	Nuclear receptor subfamily 4 group A member 1	NR4A1 or Nur77	NR4A1	P22736	-1	55	PMID: 26634653
Increased coagulation	Nuclear receptor subfamily 4 group A member 3	NR4A3 or Nor1	NR4A3	Q92570	-1	56	PMID: 26634653
Increased coagulation	Kininogen-1	KNG1	KNG1	P01042	1	57	PMID: 2553541
Increased coagulation	Plasma kallikrein	KLKB1	KLKB1	P03952	1	58	PMID: 2553541
Increased coagulation	Coagulation factor XII	F12	FA12	P00748	1	59	PMID: 2553541
Increased coagulation	Coagulation factor XI	F11	FA11	P03951	1	60	PMID: 2553541
Increased coagulation	Coagulation factor IX	F9	FA9	P00740	1	61	PMID: 2553541

Increased coagulation	Coagulation factor VIII	F8	FA8	P00451	1	62	PMID: 2553541
Increased coagulation	Coagulation factor X	F10	FA10	P00742	1	63	PMID: 2553541
Increased coagulation	Tissue factor o factor III	F3	TF	P13726	1	64	PMID: 2553541
Increased coagulation	Coagulation factor VIII	F7	FA7	P08709	1	65	PMID: 2553541
Increased coagulation	Prothrombin	F2	THRB	P00734	1	66	PMID: 2553541
Increased coagulation	Fibrinogen alpha chain	FGA	FIBA	P02671	1	67	PMID: 2553541
Increased coagulation	Fibrinogen gamma chain	FGG	FIBG	P02679	1	68	PMID: 2553541
Increased coagulation	Fibrinogen beta chain	FGB	FIBB	P02675	1	69	PMID: 2553541
Increased coagulation	Antithrombin-III	SERPINC1	ANT3	P01008	-1	70	PMID: 2553541
Increased coagulation	Tissue factor pathway inhibitor	TFPI	TFPI1	P10646	-1	71	PMID: 24620349
Increased coagulation	Angiotensinogen	AGT	ANGT	P01019	1	72	PMID: 10972684
Increased coagulation	Hyaluronidase-2	HYAL2	HYAL2	Q12891	-1	73	PMID: 25934767
Increased coagulation	Plasminogen activator inhibitor 1	SERPINE1 or PAI-1	PAI1	P05121	1	74	PMID: 8943484
Increased coagulation	Tissue-type plasminogen activator	PLAT	TPA	P00750	-1	75	PMID: 8943484
Increased coagulation	Urokinase-type plasminogen activator	PLAU	UROK	P00749	-1	76	PMID: 8943484

Supplementary Table S3. List of very common ADRs across clinical trials NCT02622321, NCT02795767, JapicCTI-121934, JapicCTI-132195, according to the definition of CIOMS1: frequency ≥ 10%.

AES number	AES name
1	Injection site reaction
2	Upper respiratory tract infection
3	Headache

Supplementary Table S4. List of drugs which evoke TMA reported in PharmaPendium.

The drugs and its corresponding targets are included as model constrains. Effect column indicates whether the drug target protein is increased/overactivated (1) or reduced/inhibited (-1) by the drug.

Number	Drug Name	Drugbank ID	Drug Target (Uniprot code)	Drug Target (Gene Name)	Effect
1	Emicizumab	DB13923	P00742	F10	1
2	aPCC	DB13151	P08709	F7	1
2	aPCC	DB13151	P00734	F2	1
2	aPCC	DB13151	P00740	F9	1
2	aPCC	DB13151	P01042	KNG1	1
2	aPCC	DB13151	P06870	KLK1	1
3	Quinine	DB00468	P16109	SELP	1
3	Quinine	DB00468	P02751	FN1	1
3	Quinine	DB00468	P12259	F5	1
3	Quinine	DB00468	P00451	F8	1
4	Cyclosporine	DB00091	Q96LZ3	PPP3R2	-1
4	Cyclosporine	DB00091	Q08209	PPP3CA	-1
4	Cyclosporine	DB00091	P16298	PPP3CB	-1
4	Cyclosporine	DB00091	P48454	PPP3CC	-1
4	Cyclosporine	DB00091	P63098	PPP3R1	-1
4	Cyclosporine	DB00091	P23284	PPIB	-1
4	Cyclosporine	DB00091	P30405	PPIF	-1
5	Tacrolimus	DB00864	P62942	FKBP1A	-1
5	Tacrolimus	DB00864	Q96LZ3	PPP3R2	-1
5	Tacrolimus	DB00864	Q08209	PPP3CA	-1
5	Tacrolimus	DB00864	P16298	PPP3CB	-1
5	Tacrolimus	DB00864	P48454	PPP3CC	-1
5	Tacrolimus	DB00864	P63098	PPP3R1	-1
6	Bevacizumab	DB00112	P15692	VEGFA	-1
7	Cocaine	DB00907	Q01959	SLC6A3	-1
7	Cocaine	DB00907	P23975	SLC6A2	-1
7	Cocaine	DB00907	P31645	SLC6A4	-1
7	Cocaine	DB00907	Q14524	SCN5A	-1
7	Cocaine	DB00907	P11229	CHRM1	-1
7	Cocaine	DB00907	P08172	CHRM2	-1
8	Docetaxel	DB01248	Q9H4B7	TUBB1	-1
8	Docetaxel	DB01248	P10415	BCL2	-1
8	Docetaxel	DB01248	P11137	MAP2	-1
8	Docetaxel	DB01248	P27816	MAP4	-1

8	Docetaxel	DB01248	P10636	MAPT	-1
9	Everolimus	DB01590	P62942	FKBP1A	-1
9	Everolimus	DB01590	P42345	MTOR	-1
10	Gemcitabine	DB00441	P04818	TYMS	-1
11	Oxaliplatin	DB00526	P04637	TP53	1
11	Oxaliplatin	DB00526	O96017	CHEK2	1
11	Oxaliplatin	DB00526	P38398	BRCA1	-1
11	Oxaliplatin	DB00526	P52701	MSH6	-1
12	Pentostatin	DB00552	P00813	ADA	-1
13	Interferon a	DB05258	P17181	IFNAR1	1
13	Interferon a	DB05258	P48551	IFNAR2	1
14	Interferon b	DB14999	P17181	IFNAR1	1
14	Interferon b	DB14999	P48551	IFNAR2	1
15	Penicillin	DB01053	P07359	GP1BA	-1
15	Penicillin	DB01053	P13224	GP1BB	-1
15	Penicillin	DB01053	P14770	GP9	-1
15	Penicillin	DB01053	P08514	ITGA2B	-1
15	Penicillin	DB01053	P05106	ITGB3	-1
15	Penicillin	DB01053	P16109	SELP	-1
16	Sirolimus	DB00877	P62942	FKBP1A	-1
16	Sirolimus	DB00877	P42345	MTOR	-1
17	Sunitinib	DB01268	P09619	PDGFRB	-1
17	Sunitinib	DB01268	P17948	FLT1	-1
17	Sunitinib	DB01268	P10721	KIT	-1
17	Sunitinib	DB01268	P35968	KDR	-1
17	Sunitinib	DB01268	P35916	FLT4	-1
17	Sunitinib	DB01268	P36888	FLT3	-1
17	Sunitinib	DB01268	P07333	CSF1R	-1
17	Sunitinib	DB01268	P16234	PDGFRA	-1
18	Vincristine	DB00541	P07437	TUBB	-1
18	Vincristine	DB00541	P68366	TUBA4A	-1
19	Mitomycin	DB00305	P05412	JUN	1
19	Mitomycin	DB00305	Q14790	CASP8	1
19	Mitomycin	DB00305	P04637	TP53	1
19	Mitomycin	DB00305	P01112	HRAS	1
20	Eculizumab	DB01257	P01031	C5	-1
21	Gemcitabine HCl (Gemcitabine)	DB00441	P04818	TYMS	-1
21	Gemcitabine HCl (Gemcitabine)	DB00441	P23921	RRM1	-1
22	Busulfan	DB01008	P42574	CASP3	1
23	Mycophenolate Mofetil	DB00688	P20839	IMPDH1	-1
23	Mycophenolate Mofetil	DB00688	P12268	IMPDH2	-1
24	Fludarabine Phosphate (Fludarabine)	DB01073	P09884	POLA1	-1
24	Fludarabine Phosphate (Fludarabine)	DB01073	P23921	RRM1	-1
25	Interferon Beta-1a	DB00060	P17181	IFNAR1	1
25	Interferon Beta-1a	DB00060	P48551	IFNAR2	1

26	Melphalan	DB01042	P42574	CASP3	1
26	Melphalan	DB01042	P19438	TNFRSF1A	1
26	Melphalan	DB01042	P20333	TNFRSF1B	1
27	Carboplatin	DB00958	P04637	TP53	1
27	Carboplatin	DB00958	O96017	CHEK2	1
27	Carboplatin	DB00958	P38398	BRCA1	-1
27	Carboplatin	DB00958	P52701	MSH6	-1
28	Oxymorphone HCl (Oxymorphone)	DB01192	P35372	OPRM1	1
28	Oxymorphone HCl (Oxymorphone)	DB01192	P41143	OPRD1	1
29	Basiliximab	DB00074	P01589	IL2RA	-1
30	Interferon beta-1b, Recombinant (Interferon beta-1b)	DB00068	P17181	IFNAR1	-1
30	Interferon beta-1b, Recombinant (Interferon beta-1b)	DB00068	P48551	IFNAR2	-1
31	Ticlopidine HCl (Ticlopidine)	DB00208	Q9H244	P2RY12	-1
32	Methylprednisolone Sodium Succinate (Methylprednisolone)	DB00959	P04150	NR3C1	1
33	Carfilzomib	DB08889	P28074	PSMB5	-1
34	Clopidogrel Bisulfate (Clopidogrel)	DB00758	Q9H244	P2RY12	-1
35	Bortezomib	DB00188	P28074	PSMB5	-1
36	Apraclonidine HCl (Apraclonidine)	DB00964	P08913	ADRA2A	1
36	Apraclonidine HCl (Apraclonidine)	DB00964	P18089	ADRA2B	1
36	Apraclonidine HCl (Apraclonidine)	DB00964	P18825	ADRA2C	1
37	Dasatinib	DB01254	P00519	ABL1	-1
37	Dasatinib	DB01254	P12931	SRC	-1
37	Dasatinib	DB01254	P29317	EPHA2	-1
37	Dasatinib	DB01254	P06239	LCK	-1
37	Dasatinib	DB01254	P07947	YES1	-1
37	Dasatinib	DB01254	P10721	KIT	-1
37	Dasatinib	DB01254	P16234	PDGFRA	-1
37	Dasatinib	DB01254	P09619	PDGFRB	-1
37	Dasatinib	DB01254	P06241	FYN	-1
37	Dasatinib	DB01254	P07948	LYN	-1
37	Dasatinib	DB01254	P08631	HCK	-1
37	Dasatinib	DB01254	P09769	FGR	-1
37	Dasatinib	DB01254	P42685	FRK	-1
37	Dasatinib	DB01254	P51451	BLK	-1
38	Doxorubicin HCl (Doxorubicin)	DB00997	P11387	TOP1	-1
38	Doxorubicin HCl (Doxorubicin)	DB00997	P11388	TOP2A	-1
39	Prednisolone	DB00860	P04150	NR3C1	1
40	Interferon Alfa-2b, Recombinant	DB00105	P17181	IFNAR1	1
40	Interferon Alfa-2b, Recombinant	DB00105	P48551	IFNAR2	1
41	Mycophenolic Acid	DB01024	P12268	IMPDH2	-1
41	Mycophenolic Acid	DB01024	P20839	IMPDH1	-1
42	Etoposide	DB00773	P11388	TOP2A	-1
42	Etoposide	DB00773	Q02880	TOP2B	-1
43	Imatinib Mesylate	DB00619	P00519	ABL1	-1

43	Imatinib Mesylate	DB00619	P10721	KIT	-1
43	Imatinib Mesylate	DB00619	P09619	PDGFRB	-1
44	Ribavirin	DB00811	P20839	IMPDH1	-1
45	Interferon Alfa-2a, Recombinant	DB00034	P17181	IFNAR1	1
45	Interferon Alfa-2a, Recombinant	DB00034	P48551	IFNAR2	1
46	Ibuprofen	DB01050	P35354	PTGS2	-1
46	Ibuprofen	DB01050	P23219	PTGS1	-1
47	Leflunomide	DB01097	Q02127	DHODH	-1
48	Rituximab	DB00073	P11836	MS4A1	-1
49	Erlotinib HCl (Erlotinib)	DB00530	P00533	EGFR	-1
50	Pazopanib HCl (Pazopanib)	DB06589	P17948	FLT1	-1
50	Pazopanib HCl (Pazopanib)	DB06589	P35968	KDR	-1
50	Pazopanib HCl (Pazopanib)	DB06589	P35916	FLT4	-1
50	Pazopanib HCl (Pazopanib)	DB06589	P16234	PDGFRA	-1
50	Pazopanib HCl (Pazopanib)	DB06589	P09619	PDGFRB	-1
50	Pazopanib HCl (Pazopanib)	DB06589	P10721	KIT	-1
50	Pazopanib HCl (Pazopanib)	DB06589	P22607	FGFR3	-1
50	Pazopanib HCl (Pazopanib)	DB06589	Q08881	ITK	-1
50	Pazopanib HCl (Pazopanib)	DB06589	P05230	FGF1	-1
50	Pazopanib HCl (Pazopanib)	DB06589	P07333	CSF1R	-1
50	Pazopanib HCl (Pazopanib)	DB06589	P06239	LCK	-1
51	Tranexamic Acid	DB00302	P00747	PLG	-1
52	Daclizumab	DB00111	P01589	IL2RA	-1
53	Pemetrexed Disodium (Pemetrexed)	DB00642	P04818	TYMS	-1
53	Pemetrexed Disodium (Pemetrexed)	DB00642	P00374	DHFR	-1
53	Pemetrexed Disodium (Pemetrexed)	DB00642	P22102	GART	-1
54	Levofloxacin	DB01137	P11388	TOP2A	-1
55	Bosentan	DB00559	P24530	EDNRB	-1
55	Bosentan	DB00559	P25101	EDNRA	-1
56	Capecitabine	DB01101	P04818	TYMS	-1
57	Ranibizumab	DB01270	P15692	VEGFA	-1
58	Trastuzumab	DB00072	P04626	ERBB2	-1
59	Cytarabine	DB00987	P06746	POLB	-1
60	Alendronate Sodium (Alendronic acid)	DB00630	P14324	FDPS	-1
61	Azathioprine	DB00993	O95602	POLR1A	-1
61	Azathioprine	DB00993	P24928	POLR2A	-1
62	Ciprofloxacin HCl (Ciprofloxacin)	DB00537	P11388	TOP2A	-1
62	Ciprofloxacin HCl (Ciprofloxacin)	DB00537	Q02880	TOP2B	-1
63	Infliximab	DB00065	P01375	TNF	-1
64	Levonorgestrel	DB00367	P06401	PGR	1
64	Levonorgestrel	DB00367	P03372	ESR1	1
65	Sorafenib Tosylate (Sorafenib)	DB00398	P15056	BRAF	-1
65	Sorafenib Tosylate (Sorafenib)	DB00398	P04049	RAF1	-1

65	Sorafenib Tosylate (Sorafenib)	DB00398	P35916	FLT4	-1
65	Sorafenib Tosylate (Sorafenib)	DB00398	P35968	KDR	-1
65	Sorafenib Tosylate (Sorafenib)	DB00398	P17948	FLT1	-1
65	Sorafenib Tosylate (Sorafenib)	DB00398	P36888	FLT3	-1
65	Sorafenib Tosylate (Sorafenib)	DB00398	P09619	PDGFRB	-1
65	Sorafenib Tosylate (Sorafenib)	DB00398	P10721	KIT	-1
65	Sorafenib Tosylate (Sorafenib)	DB00398	P07949	RET	-1
66	Diclofenac Sodium	DB00586	P35354	PTGS2	-1
66	Diclofenac Sodium	DB00586	P23219	PTGS1	-1
67	Fluorouracil	DB00544	P04818	TYMS	-1
68	Gemtuzumab Ozogamicin	DB00056	P09874	PARP1	1
69	Omeprazole	DB00338	P20648	ATP4A	-1
70	Peginterferon Alfa-2a	DB00008	P48551	IFNAR2	1
70	Peginterferon Alfa-2a	DB00008	P17181	IFNAR1	1
71	Paclitaxel	DB01229	Q9H4B7	TUBB1	-1
71	Paclitaxel	DB01229	P10415	BCL2	-1
71	Paclitaxel	DB01229	Q13509	TUBB3	-1
71	Paclitaxel	DB01229	P11137	MAP2	-1
71	Paclitaxel	DB01229	P27816	MAP4	-1
71	Paclitaxel	DB01229	P10636	MAPT	-1

Supplementary Table S5. TPMS training set.

Summary of data (number of entries in the database for each data type) used for model construction (network and training set).

Entry type	# Entries
In-house databases information	
Considered Interactions	312.445
Considered Proteins	15.555
Characterized Drugs	4.729
Drug Targets	2.125
Characterized Clinical Conditions	222
Clinical Conditions Key Proteins Characterized	3.712
Truth table information	
Curated drug-indications restrictions	169,335 (1,961 positive)
Drug-ADRs restrictions	19,350 (573 positive)
Drug-indications/ADRs protein correlations	2.175

Supplementary Table S6. Summary of the sources of information found in the scientific literature supporting the predicted mechanisms for emicizumab plus rFVIIa and emicizumab plus aPCC. Interactions between Node A to Node B.

Link number	Node A		Node B		Reference
	Uniprot code	Gene Name	Uniprot code	Gene Name	
1	Emicizumab+ aPCC		P00734	F2	Drug target or drug component
2	Emicizumab+ aPCC		P00740	F9	Drug target or drug component
3	Emicizumab+ aPCC		P00742	F10	Drug target or drug component
4	Emicizumab+ aPCC		P01042	KNG1	Drug target or drug component
5	Emicizumab+ aPCC		P08709	F7	Drug target or drug component
6	Emicizumab+ aPCC		P00742	F10	Drug target or drug component
7	Emicizumab+ aPCC		P08709	F7	Drug target or drug component
8	P00734	F2	P00740	F9	PMID 1652157
9	P00734	F2	P02671	FGA	PMID 15572239
10	P00734	F2	P02675	FGB	PMID 15572239
11	P00734	F2	P02679	FGG	PMID 15572239
12	P00734	F2	P03951	F11	PMID 1652157
13	P00734	F2	P07359	GP1BA	PMID 15381249
14	P00734	F2	P14770	GP9	PMID 15381249
15	P00734	F2	P40197	GP5	PMID 15381249
16	P00734	F2	TMA		PMID: 2553541
17	P00740	F9	P00742	F10	KEGG: 04610-Complement and coagulation cascades; PMID 9218428
18	P00740	F9	TMA		PMID: 2553541
19	P00742	F10	P00734	F2	PMID 17208341;KEGG: 04610-Complement and coagulation cascades
20	P00742	F10	TMA		PMID: 2553541
21	P01042	KNG1	TMA		PMID: 2553541
22	P02671	FGA	P02675	FGB	PMID: 9333233
23	P02671	FGA	P02679	FGG	PMID: 9333233
24	P02671	FGA	TMA		PMID: 2553541
25	P02675	FGB	P02671	FGA	PMID: 9333233
26	P02675	FGB	P02679	FGG	PMID: 9333233
27	P02675	FGB	TMA		PMID: 2553541
28	P02679	FGG	P02671	FGA	PMID: 9333233
29	P02679	FGG	P02675	FGB	PMID: 9333233
30	P02679	FGG	TMA		PMID: 2553541
31	P03951	F11	P00740	F9	PMID 2019570
32	P03951	F11	TMA		PMID: 2553541
33	P07359	GP1BA	P00734	F2	PMID: 11696542

34	P07359	GP1BA	P03951	F11	PMID: 11696542
35	P07359	GP1BA	P14770	GP9	PMID: 2436691
36	P07359	GP1BA	P40197	GP5	PMID: 1730602
37	P07359	GP1BA	TMA		PMID 15381249
38	P08709	F7	P00740	F9	PMID: 2248955
39	P08709	F7	P00742	F10	PMID 17475978
40	P08709	F7	TMA		PMID: 2553541
41	P14770	GP9	P00734	F2	PMID: 11696542
42	P14770	GP9	P03951	F11	PMID: 11696542
43	P14770	GP9	P07359	GP1BA	PMID: 2436691
44	P14770	GP9	P40197	GP5	PMID: 23336709; PMID: 23336710
45	P14770	GP9	TMA		PMID 15381249
46	P40197	GP5	P00734	F2	PMID: 11696542
47	P40197	GP5	P03951	F11	PMID: 11696542
48	P40197	GP5	P07359	GP1BA	PMID: 1730602
49	P40197	GP5	P14770	GP9	PMID: 23336710
50	P40197	GP5	TMA		PMID 15381249
